# Reward Prediction-Error Fidelity: a testable hypothesis linking reinforcement learning mechanisms to applied behavior analysis for autism and neurodevelopmental conditions

**DOI:** 10.3389/fnbeh.2026.1974577

**Published:** 2026-10-27

**Authors:** Sebastian G. D. Cesarano

**Affiliations:** SiPGI, Postgraduate School of Integrated Gestalt Psychotherapy, Torre Annunziata, Naples, Italy

**Keywords:** applied behavior analysis, autism, credit assignment, eligibility traces, hypothesis and theory, reinforcement learning, reward prediction error

## Abstract

Applied behavior analysis (ABA) has developed an effective technology of reinforcement delivery, including Discrete Trial Training, Natural Environment Teaching, and token economies, largely on functional-behavioral grounds. Computational neuroscience has independently produced formal accounts of reinforcement learning centered on prediction errors and credit assignment. I propose a testable hypothesis connecting the two: the effectiveness of a reinforcement procedure depends on the quality of the learning signal that its delivery produces on each occasion, a property I term Reward Prediction-Error Fidelity (RPE Fidelity). The property is decomposed into four dimensions, temporal credit, contingency, prediction, and outcome value, each corresponding to a construct long studied in behavior analysis. Formalized within an actor-critic model with eligibility traces, the four dimensions enter the learning update multiplicatively, which implies that they cannot compensate for one another and that the effect of each depends on the others and on the learner’s history. The combination rule distinguishes the hypothesis from the separate behavioral constructs it draws on, and its dependence on learning history distinguishes it from steady-state quantitative models of reinforcement. I situate the hypothesis within an integrative framework, discuss its application to autistic learners, including differences in reward processing and volatility estimation and the role of learner assent, and compare it with procedural fidelity, quantitative behavioral theory, and alternative learning models, including attentional, timescale-invariant, successor-representation, and Bayesian accounts. I derive nine predictions, specifying for each the result that would favor the hypothesis and the result that would favor its strongest competitor, and outline a staged research programme compatible with single-case experimental designs.

## 1 Introduction

Reinforcement is one of the few constructs studied with comparable rigor at both the neurobiological and the behavioral level of analysis, yet the dialogue between these two literatures remains surprisingly limited in practice. Neuroscience has, over the past three decades, produced a detailed mechanistic account of how the brain represents and updates value, from the discovery of the dopaminergic reward prediction error to the cellular dynamics of eligibility traces and synaptic plasticity. Independently, applied behavior analysis (ABA) has developed, refined, and empirically validated a technology of reinforcement delivery (encompassing procedures such as Discrete Trial Training (DTT), Natural Environment Teaching (NET), and token economy systems) almost entirely on functional-behavioral grounds, with comparatively little explicit reference to the underlying neurobiology.

This separation is not merely an academic curiosity: it means that many long-standing ABA practice principles (the immediacy of reinforcement, the differential durability of intermittent versus continuous schedules, the value of pairing tangible and social reinforcers) are empirically well supported but rarely explained in terms of the mechanisms that produce them. Conversely, neurobiological findings on reward learning rarely engage with the applied, real-world contingency management systems in which reinforcement is most systematically and repeatedly deployed. This article addresses that gap by proposing a specific, testable hypothesis; it does not attempt an exhaustive review.

I propose the following hypothesis. The effectiveness of a reinforcement procedure depends on the quality of the learning signal produced by each delivery, beyond the identity of the reinforcer and adherence to the written protocol. I term this property Reward Prediction-Error Fidelity (RPE Fidelity) and decompose it into four dimensions: temporal credit, contingency, prediction, and outcome value. Each of these variables corresponds to a construct that behavior analysis has long studied, namely immediacy of reinforcement, response-reinforcer contingency, schedule arrangement, and motivating operations. The claim specific to this article concerns how they combine. When the four dimensions are formalized as terms of a single learning update (Section 2.3), they enter multiplicatively rather than additively. This implies that they cannot compensate for one another: a severe deficit in any one of them limits learning regardless of the others, and the effect of improving one depends on the level of the rest. The combination rule distinguishes RPE Fidelity from the separate behavioral constructs it draws on, its dependence on the learner’s history distinguishes it from steady-state quantitative models of reinforcement, and together they make the hypothesis testable in a discriminating way (Section 7.2). RPE Fidelity is also distinct from procedural fidelity: a procedure can be delivered exactly as written while producing a degraded learning signal.

The argument proceeds from neural and computational foundations to applied reinforcement procedures, competing models, and discriminating empirical predictions.

## 2 Theoretical background: neural foundations of reinforcement

### 2.1 Dopaminergic reward circuitry

The neurobiological basis of reinforcement is largely attributed to phasic activity in midbrain dopamine neurons, particularly within the ventral tegmental area (VTA), which project to the nucleus accumbens (NAc) and prefrontal cortex. The seminal work of Schultz et al. (1997) established that these neurons do not simply signal the occurrence of reward, but rather encode a reward prediction error (RPE): a bidirectional signal reflecting the discrepancy between expected and received outcomes. Dopamine neurons are activated above baseline when reward exceeds expectation, remain unchanged when reward is fully predicted, and are suppressed below baseline when an expected reward is omitted.

This RPE signal has since been characterized as a biologically plausible teaching signal, capable of adjusting synaptic strength until an organism’s internal estimate of value converges with actual outcomes (Glimcher, 2011). More recent work has complicated this picture without overturning it: dopamine neurons show functional heterogeneity, and a purely scalar, unidimensional account of the RPE signal appears insufficient to capture the full range of dopaminergic responses observed across tasks and brain regions (Nasser et al., 2017; Kahnt and Schoenbaum, 2025; Gershman et al., 2024). Recent single-neuron recordings add a further complication: at least part of what was traditionally interpreted as a pure learning signal may instead reflect ongoing regulation of behavioral performance and motor vigor, indicating that distinct dopaminergic subpopulations may encode parallel value functions (Bakhurin et al., 2025). Alternative accounts go further, proposing that mesolimbic dopamine conveys causal associations between events rather than prediction errors (Jeong et al., 2022; see Section 6.10). The present hypothesis therefore requires only that the RPE constitute one important computational mechanism through which dopaminergic signaling contributes to reinforcement learning. It does not require a unitary account of dopamine function.

### 2.2 Computational models of reinforcement

The RPE hypothesis derives much of its explanatory power from its correspondence with formal computational models of learning developed independently in psychology and machine learning. The Rescorla and Wagner (1972) model first formalized the notion that learning is driven by the discrepancy between predicted and actual reinforcement, a principle later generalized by temporal-difference (TD) reinforcement learning algorithms (Sutton and Barto, 2018), which extend this logic across sequences of states and predictions rather than single associative events. The convergence between the firing patterns of dopamine neurons and the error term of TD models is widely regarded as one of the more notable points of contact between computational neuroscience and behavioral theory, while the field continues to debate how completely this convergence generalizes given documented dopaminergic heterogeneity (Section 2.1); it nonetheless provides a principled, if partial, bridge between operant learning as conceptualized in ABA and its underlying neural implementation.

### 2.3 A formal account: actor-critic learning with eligibility traces

Operant learning requires the learner to estimate not only the value of situations but also which response to emit in them. I therefore formalize the hypothesis within an actor-critic architecture (Barto et al., 1983; Sutton and Barto, 2018), which has been proposed as a model of the functional division between ventral and dorsal striatum (Joel et al., 2002; O'Doherty et al., 2004). The critic learns state values *V*(*s*) from the temporal-difference prediction error:


$$\delta_{t}=r_{t}+\gamma \cdot V(s_{t+1})-V(s_{t})$$ (1)

The actor maintains response preferences *H*(*s*,*a*), which determine the probability of each response through a softmax policy, and updates them using the same error:


$$H(s,a)\leftarrow H(s,a)+\alpha_{A}\cdot \delta_{t}\cdot e_{t}(s,a)$$ (2)

where *e*_t_(*s*,*a*) is an eligibility trace marking recently emitted responses as candidates for credit:


$$e_{t}(s,a)=\gamma \lambda \cdot e_{t-1}(s,a)+1[s_{t}=s,a_{t}=a]$$ (3)

with *λ* ∈ [0,1] governing how quickly eligibility decays [TD(*λ*); Sutton and Barto, 2018]. A formulation based on action values *Q*(*s*,*a*), such as SARSA(*λ*), leads to the same qualitative conclusions. Eligibility traces in this algorithmic sense are distinct from, but have been proposed to correspond to, the synaptic tagging processes described in Section 3.1 (Gerstner et al., 2018).

Consider a single teaching trial in which the target response a^*^ is emitted in state *s* and followed, after *k* time steps, by the first stimulus that reliably signals reward, and then by the outcome *o*. For simplicity, the outcome is treated as terminating the trial, so that *V*(*s*_t + 1_) = 0. The four dimensions of RPE Fidelity then correspond to specific terms of the model.

#### 2.3.1 Temporal credit

When the error is computed, the eligibility of a^*^ is (*γλ*)^k^. Temporal Credit Fidelity is therefore defined by *k*, the delay between the target response and the first reliable reward signal, rather than by the delay to the primary reinforcer. This distinction is central in applied settings. Once a stimulus such as praise, a token, or a marker has acquired value through pairing, its onset itself generates a prediction error [*δ* = *γ*·*V*(*s*_marker_) − *V*(*s*_prev_)], so that conditioned reinforcers can bridge delays far longer than the cellular window described in Section 3.1 (Williams, 1994). The window of approximately 0.3–2 s therefore constrains, at the synaptic level, the interval between the response and the first reliable value signal, not the interval between the response and a backup reinforcer.

#### 2.3.2 Outcome value

The reward term is *r* = *u*(*o*)·*m*, where *u*(*o*) is the unconditioned value of the outcome and *m* ∈ [0,1] is the learner’s current motivational state with respect to it. Motivating operations (Laraway et al., 2003) act on *m*: satiation and habituation lower it, deprivation raises it. Outcome Value Fidelity is thus defined by the product *u*(*o*)·*m* at the moment of delivery, not by nominal reinforcer preference.

#### 2.3.3 Contingency

Outcomes that occur in the absence of the target response are learned by the critic as value of the context *c*, which forms part of *V*(*s*_t_) at the time of the outcome. Because the error following the target response is computed against this baseline, *δ* = *r* − *V*(*c*), noncontingent outcomes that raise *V*(*c*) reduce the error that follows a*, and therefore reduce the actor’s update. Contingency accordingly acts through the value of the context, not only through the representation of the response. In the Rescorla-Wagner model with the context treated as a competing cue, asymptotic associative strength corresponds to Δ*P* = *P*(*O*|*R*) − *P*(*O*|¬*R*) under the conditions analyzed by Chapman and Robbins (1990), which provides a formal link between Contingency Fidelity and the classic contingency literature on operant behavior (Rescorla, 1968; Hammond, 1980).

#### 2.3.4 Prediction

Prediction Fidelity comprises two quantities that must be kept distinct. The first is the signed prediction error δ itself: an outcome that is fully predicted produces no error and supports no further learning, whatever its value. The second is associability, the rate at which the learner updates in response to a given error, which attentional models tie to recent unsigned error (Pearce and Hall, 1980):


$$\alpha_{t}=\eta \cdot |\delta_{t-1}|+(1-\eta )\cdot \alpha_{t-1}$$ (4)

The uncertainty-related dopaminergic signal described by Fiorillo et al. (2003) is more closely aligned with this associability term than with *δ* (Section 4.1). Prediction Fidelity therefore has two components: whether the delivery leaves the outcome informative given current expectation, and whether the schedule sustains associability appropriate to the current stage of learning. Replacing the fixed learning rate in Equation 2 with *α_A_*·*α_t_* yields a hybrid formulation of the kind proposed by Le Pelley (2004).

Substituting these terms, the update to the preference for the target response at the time of the outcome is:


$$\mathrm{\Delta H}(s,a^{*})=\alpha_{A}\cdot \alpha_{t}\cdot [u(o)\cdot m-V(c)]\cdot {\left(\gamma \lambda \right)}^{k}$$ (5)

Equation 5 describes the update on a single reinforced trial. Averaging over emissions of the target response that are and are not followed by the outcome, and assuming that the value of the context reflects noncontingent outcomes of the same kind and value, *V*(*c*) = *P*(*O*|¬*R*)·*u*(*o*)·*m*, the expected update per emission of the target response is:


$$E[\mathrm{\Delta H}(s,a^{*})]=\alpha_{A}\cdot \alpha_{t}\cdot {\left(\gamma \lambda \right)}^{k}\cdot u(o)\cdot m\cdot \mathrm{\Delta P}$$ (6)

In this form, all four dimensions appear as factors of a single product: temporal credit through (*γλ*)*^k^*, outcome value through *u*(*o*)·*m*, contingency through Δ*P*, and prediction through *α_t_* and through the dependence of the realized error on the learner’s current expectation. The derivation makes its assumptions explicit. The product form holds when noncontingent outcomes are of the same kind and value as contingent ones and are not signaled by a distinct stimulus; when they are signaled, *V*(*c*) does not rise and the contingency factor tends toward *P*(*O*|*R*), which is the basis of Prediction 2. Three consequences follow from Equations 5, 6.

First, non-compensation. The dimensions enter Equation 6 as factors of a product, not as terms of a sum. If any factor approaches zero [a delay long enough that (*γλ*)*^k^* ≈ 0, a context that already predicts the outcome as well as the response does, a fully satiated learner, or exhausted associability], the update approaches zero regardless of the other factors. At the level of the learning update, the combination rule is therefore multiplicative; whether the observable ratings of the profile (Section 6.8) show the same structure remains an empirical question, because the mapping from dimensions to ratings is itself imperfect.

Second, interaction. Because the dimensions multiply, the effect of improving one depends on the level of the others; the cross-partial derivatives of Equation 5 are nonzero. For example, increasing outcome value should produce a smaller gain when temporal credit is poor than when it is good. This property of the model is tested directly by Prediction 1.

Third, history dependence. *V*(*c*) and *α_t_* change with the learner’s history, so identical delivery conditions produce different updates at different stages of learning. As expected value approaches *u*(*o*)·*m*, *δ* shrinks; this is why Prediction Fidelity is defined relative to the stage of learning, and why the same reduction in predictability can impair acquisition yet support maintenance (Prediction 3).

#### 2.3.5 Status of the multiplicative structure

The multiplicative structure of Equations 5, 6 is not specific to the actor-critic formulation. A product of an error term, an eligibility or salience term, and a learning rate is shared by error-driven learning rules generally, from the Rescorla and Wagner (1972) model, in which the prediction error is scaled by salience parameters of the stimulus and the outcome, to three-factor synaptic learning rules (Gerstner et al., 2018). The combination rule is therefore a robust property of the family of models on which the prediction-error account rests, not a consequence of a particular modelling choice. The hypothesis does not claim to introduce a new learning algorithm. Its claim is that the variables applied behavior analysis manipulates on each delivery are the arguments of this product, so that a structural property of error-driven learning becomes an empirical constraint on applied procedures, where it has not been tested and where practice recommendations typically address timing, contingency, schedule, and reinforcer value as separate considerations. Where the present formulation does depend on specific modelling choices, namely the absolute scaling of the eligibility trace, the role of context value in contingency, and the history dependence carried by *V*(*c*) and *α_t_*, these choices generate the predictions that distinguish it from alternative accounts (Predictions 2, 3, and 6–9).

#### 2.3.6 Relation to existing behavioral constructs

Each dimension corresponds to a construct already studied in behavior analysis: delay of reinforcement (Lattal, 2010), response-reinforcer contingency (Rescorla, 1968; Hammond, 1980), schedule arrangement (Ferster and Skinner, 1957), and motivating operations (Laraway et al., 2003). These constructs specify the effect of each variable, but not how the variables combine, nor how their effects change with learning history; Equation 5 specifies both. The account also differs from Killeen’s (1994) Mathematical Principles of Reinforcement, which likewise has a multiplicative structure and whose coupling coefficient plays a role analogous to the eligibility trace. The difference lies in the third consequence: the Mathematical Principles of Reinforcement primarily describe steady-state response rates, whereas Equation 5 describes trial-by-trial acquisition and contains terms, *V*(*c*) and *α_t_*, through which learning history modulates the effect of identical delivery conditions. The discriminating tests between the two accounts therefore concern learning trajectories rather than asymptotic performance (Section 7.2).

#### 2.3.7 Model-free and goal-directed control

Equations 1–5 describe model-free learning. Sensitivity to contingency degradation and to outcome devaluation are, however, classic signatures of goal-directed control (Balleine and Dickinson, 1998), and instrumental behavior is thought to reflect an arbitration between the two systems that shifts with training (Daw et al., 2005). The hypothesis does not require that ABA procedures be learned exclusively through model-free computation; it requires only that the delivery conditions captured by the four dimensions determine the quality of the experience from which both systems learn. The two systems nonetheless make different predictions about Outcome Value Fidelity, which provides an additional test. Under goal-directed control, a change in *m* (for example, satiation before a session) should alter responding immediately, before the devalued outcome is experienced; under model-free control, it should alter responding only after the outcome has been experienced and the error updated. Because goal-directed control tends to predominate early in training and habitual control after extended practice, this difference should itself depend on the stage of learning.

#### 2.3.8 Scope

The formal account specifies what would have to be true of the underlying computation for the hypothesis to hold; it does not show that ABA procedures are implemented by this computation. Alternative learning models, including attentional, Bayesian, successor-representation, and retrospective causal accounts, treat some of the same dimensions differently and are compared with the present formulation in Section 6.10.

## 3 Reinforcement timing and synaptic consolidation

### 3.1 The synaptic eligibility trace

A central question in translating dopaminergic mechanisms into applied practice concerns the temporal window within which reinforcement remains behaviorally effective. At the cellular level, this question has been addressed through the concept of a synaptic eligibility trace. Using optogenetic stimulation of dopaminergic and glutamatergic inputs separately in striatal medium spiny neurons, Yagishita et al. (2014) showed that dopamine promotes spine enlargement only within a narrow window, approximately 0.3–2 s after the preceding glutamatergic input, with the precise boundaries of this window set by the kinetics of cyclic AMP regulation in thin distal dendrites. This work, together with the behavioral correlate described by Yamaguchi et al. (2022), provides a candidate cellular mechanism consistent with the well-established behavioral principle that reinforcement is most effective when delivered promptly after the target response, while also showing that this window is a range with a specific molecular basis rather than a single fixed threshold. As formalized in Section 2.3, this window constrains the interval between a response and the first stimulus that reliably signals reward, which in applied settings is frequently a conditioned reinforcer rather than the primary outcome.

This finding offers a candidate mechanism for what applied behavior analysts have long formalized as the immediacy principle: reinforcement delivered without delay following a target response is, in many paradigms, associated with stronger and more reliable learning than delayed reinforcement, a pattern consistent with the eligibility-trace window described above. As Section 3.2 details, this should not be read as a fixed biological threshold that transfers directly to instruction; the size of the delay effect depends on whether the learner has other information supporting correct attribution of the outcome to the response.

### 3.2 Behavioral consequences of reinforcement delay

Complementing this cellular evidence, behavioral studies in humans confirm that the timing of reinforcement shapes not only the rate of acquisition but also the durability of what is learned. In a motor-skill learning task, delaying reward delivery by a few seconds altered the trajectory of learning: training with a short delay (1 s) produced continuous improvement in performance, whereas a long delay (6 s) produced high initial learning rates followed by an early plateau, lower performance at the end of training, and reduced overnight consolidation (Vassiliadis et al., 2022). The authors motivated this comparison with earlier neuroimaging evidence that short and long reward delays preferentially engage fronto-striatal and hippocampal systems, respectively; the study itself did not measure neural activity, so this dissociation remains a plausible interpretation rather than a demonstrated mechanism.

These findings should not, however, be read as establishing a fixed, universal delay threshold that transfers directly from single-synapse recordings to classroom instruction. A substantial literature on human causal learning shows that the detrimental effect of a response-outcome delay depends heavily on whether the learner has structural knowledge of the relevant timeframe: Buehner and May (2003) found that providing cues to the trial structure significantly improved causal judgments in the presence of delay, and a follow-up study showed that the negative effect of delay on causal learning could be abolished entirely by a change in instructions alone (Buehner and May, 2004). These studies measured explicit causal judgments in adults, and whether the same effect holds for implicit operant learning, particularly in children who cannot report their expectations about timing, remains an open question. Analogous findings from the behavior-analytic literature on delay-of-reinforcement gradients (Lattal, 2010) and temporal discounting (Critchfield and Kollins, 2001), which do concern operant behavior directly, show that delay effects are well documented independently of any neurobiological account. Together, this evidence indicates that temporal contiguity and correct credit assignment are related but dissociable: a response and its consequence can be separated by several seconds and still be attributed correctly if the learner has adequate information about the response-outcome relationship. I therefore treat reinforcement timing not as a fixed biological threshold to be imported directly into instructional design, but as one input, among others, to the temporal credit assignment problem formalized in Section 2.3.

### 3.3 Implications for DTT and NET

Discrete Trial Training (DTT) typically pairs a discriminative stimulus, response, and reinforcer within a tightly controlled and immediate sequence, while Natural Environment Teaching (NET) often involves greater variability in the interval between response and reinforcement due to its embeddedness in naturalistic activity (Box 1). Rather than treating this as evidence that DTT is intrinsically better aligned with the eligibility-trace window than NET, which is not a necessary property of either technology, the more precise question is how well timing and contingency are controlled within each method on a given occasion. Framed this way, RPE Fidelity becomes a property of how a procedure is implemented on a specific trial, independent of which ABA technology is used.

BOX 1 **Discrete Trial Training and Natural Environment Teaching**

*Discrete Trial Training (DTT)* is an adult-led instructional format in which teaching is divided into brief, repeated trials, each consisting of a discriminative stimulus presented by the instructor, the learner’s response, a prompt when needed, a consequence, and a short intertrial interval (Smith, 2001). Trials are typically delivered in a structured setting, with reinforcers often selected through prior preference assessment and delivered immediately after correct responses. Because the sequence of events is tightly controlled, DTT tends by design to support Temporal Credit and Contingency Fidelity. Its characteristic risks concern the other two dimensions: when the same reinforcer follows every correct response across many trials, the outcome becomes fully predictable (Prediction Fidelity) and may lose value through satiation (Outcome Value Fidelity).

*Natural Environment Teaching (NET)* embeds teaching opportunities in activities that the learner initiates or is already engaged in, such as play or daily routines. The instructor uses the learner’s current motivation to create a learning opportunity, and the reinforcer is usually a natural consequence of the target response itself: a request for a ball, for example, is reinforced by access to the ball (Cooper et al., 2020). Naturalistic approaches of this kind are now grouped, together with developmental approaches, under the heading of naturalistic developmental behavioral interventions (Schreibman et al., 2015). Because the learner’s motivation is captured rather than arranged, NET tends to support Outcome Value Fidelity, and the variability of natural contexts supports generalization. Its characteristic risks are the reverse of those of DTT: the interval between response and consequence, and the clarity of the relation between them, depend on how the instructor handles each occasion, so that Temporal Credit and Contingency Fidelity vary more from one opportunity to the next.

In practice, most programmes combine the two formats. Within the present framework, the relevant distinction is not between methods but between occasions: either format can be delivered with high or low fidelity on each of the four dimensions.

## 4 Reinforcement schedules and educational applications

### 4.1 From continuous to intermittent reinforcement

Once a target behavior has been established, the schedule by which reinforcement is delivered becomes a critical determinant of both its strength and its resistance to extinction, as established in the classic behavior-analytic literature on schedules of reinforcement (Ferster and Skinner, 1957). Continuous reinforcement (in which every instance of the target behavior is reinforced) produces rapid initial acquisition but yields responding that extinguishes quickly once reinforcement is withdrawn. Intermittent schedules, by contrast, particularly variable-ratio (VR) arrangements in which reinforcement follows an unpredictable number of responses, generate higher and more sustained rates of responding that are considerably more resistant to extinction.

This behavioral asymmetry, known as the partial reinforcement extinction effect, has well-established behavioral explanations, including frustration theory (Amsel, 1962) and sequential theory (Capaldi, 1966), which attribute it, respectively, to the conditioning of responding in the presence of anticipatory frustration and to learned associations between sequences of nonreinforced responses and subsequent reinforcement. Neurobiological findings are compatible with these accounts but do not replace them. Phasic dopamine responses to reward-predicting stimuli vary monotonically with reward probability, whereas a slower, sustained component of activity has been reported to covary with outcome uncertainty, which is maximal at a probability of 0.5 (Fiorillo et al., 2003). The interpretation of this sustained component remains debated: it has been argued that it may arise from averaging, across trials, prediction errors whose positive and negative values are coded asymmetrically, rather than from a distinct uncertainty signal (Niv et al., 2005). Under either interpretation, the finding bears on the associability component of Prediction Fidelity (Section 2.3) rather than on the prediction error itself, and it concerns the informativeness of reinforcement rather than unpredictability as such; it should not be read as implying that unpredictability should be maximized (Section 6.4).

### 4.2 Translating reinforcement schedules to the classroom

A pilot study in an elementary school classroom embedded variable-ratio contingencies within a token-based game, with the explicit aim of eliciting reward prediction errors, and reported increased student engagement (Eckert et al., 2023). The study did not measure prediction-error signals, so its results are compatible with, but do not demonstrate, the involvement of the mechanism proposed here.

More broadly, the token economy, in which tokens earned for target behaviors are later exchanged for backup reinforcers, is among the most extensively evaluated reinforcement-based classroom strategies. Meta-analyses report large effects in general and special education classrooms, moderated by components such as backup reinforcer type and exchange schedule (Kim et al., 2022; Soares et al., 2016), although an evaluation against What Works Clearinghouse standards concluded that methodological weaknesses in the single-case literature prevented classifying token economies as a best practice (Maggin et al., 2011).

The practical relevance of this evidence is sharpened by a systematic review of 96 published token-economy studies, which found that only 19% (18 of 96 articles) provided replicable, complete descriptions of all primary procedural components (Ivy et al., 2017). Token economies are known to be effective on average, but the specific configuration of their parameters, the token production schedule, the exchange schedule, and backup reinforcer characteristics, is often left underspecified in the published literature. This is precisely the gap that a dimensional account such as RPE Fidelity is positioned to address: rather than asking only whether a token economy was used, it asks which specific configuration of timing, contingency, predictability, and value parameters was in place.

Beyond token economies, differential reinforcement procedures, in which reinforcement is delivered contingent on a specific, targeted response class while other responses are placed on extinction, represent a second major family of reinforcement-based classroom interventions. A recent systematic literature review of differential reinforcement in applied settings for individuals with autism found large to very large effects across seventeen studies, with differential reinforcement of alternative behavior and of low rates of behavior producing particularly strong outcomes (Muharib and Walker, 2025). A complementary systematic review focused specifically on skill acquisition, rather than behavior reduction, found that differential reinforcement conditions favoring independent responding over prompted responding produced faster skill acquisition across the studies reviewed (Cividini-Motta et al., 2024). Together, these findings extend the evidence base for reinforcement scheduling beyond token economies to the broader family of differential reinforcement procedures used routinely in DTT and NET.

Meta-analytic evidence supports the effectiveness of Early Intensive Behavioral Intervention for young autistic children, although the scarcity of randomized designs introduces uncertainty into these estimates (Eldevik et al., 2009, 2026; Reichow et al., 2018; Han et al., 2026). Because the present hypothesis concerns the parameters of reinforcement delivery rather than the effectiveness of comprehensive programmes, studies that manipulate individual reinforcement parameters, such as those reviewed above, are more directly relevant to testing it.

## 5 Boundary conditions and motivational moderators

The evidence in this section bears on the Outcome Value and Prediction dimensions: it concerns the conditions under which the value of a reinforcer, and of the activity it follows, changes as a result of reinforcement itself.

### 5.1 The overjustification effect

The same reward circuitry that supports reinforcement-based learning can, under certain conditions, undermine motivation once extrinsic reinforcement is withdrawn, a phenomenon described as the overjustification effect. When performance-based monetary reward was introduced for an intrinsically engaging task and subsequently removed, activity in the anterior striatum and prefrontal regions declined in parallel with voluntary task engagement (Murayama et al., 2010). A meta-analysis of 128 studies found that tangible rewards contingent on engagement, completion, or performance undermined free-choice intrinsic motivation, more strongly in children than in college students (Deci et al., 1999; see also Tang and Hall, 1995), although later syntheses show that incentives and intrinsic motivation jointly predict performance and that the effect depends on how directly the incentive is tied to the behavior measured (Cerasoli et al., 2014).

Two findings qualify the relevance of this literature for applied behavior analysis. First, a quantitative review of 65 data sets from individuals with intellectual and developmental disabilities found a mean effect that did not differ from zero, with outcomes consistent with overjustification only somewhat more likely for behaviors already occurring at high levels without extrinsic reinforcement (Levy et al., 2017); the risk therefore concerns mainly behaviors that already occur spontaneously, which bears on target selection rather than on the use of reinforcement as such. Second, the type of reinforcer appears to matter: in a recent functional imaging study, both monetary and social rewards preserved neural intrinsic value after withdrawal, but only monetary reward reduced behavioral motivational drive (Ayabe et al., 2026). This dissociation offers a partial rationale for the common practice of pairing tangible reinforcers with social praise, and bears directly on the treatment of social reward in Section 6.5.

### 5.2 Reinforcement unpredictability as a boundary condition

The same unpredictability that makes variable-ratio schedules effective for establishing durable, extinction-resistant behavior (Section 4.1) is mechanistically related to reinforcement dynamics documented in the behavioral addiction literature, where both the variability and frequency of reward delivery contribute to the addictive potential of a reinforcer (Clark and Zack, 2023). This is not a reason to avoid variable-ratio schedules in ABA practice, which remain well supported, but a reason to apply them deliberately and with planned fading once a skill is consolidated, rather than sustaining high variability indefinitely.

## 6 The hypothesis: learning-signal quality and Reward Prediction-Error Fidelity

### 6.1 Learning-signal quality: a unifying principle

The evidence reviewed in Sections 2–5 spans cellular, computational, behavioral, and instructional levels of explanation. I organize it around a single principle: a reinforcement event supports learning to the extent that its delivery allows the learner to answer four questions. Two concern credit assignment in the strict sense: what produced the outcome (contingency), and when the relevant response occurred (temporal credit). Two concern the computations that operate on that attribution: how much the outcome differs from what was expected (prediction), and how much it is worth at that moment (outcome value). I refer to the degree to which delivery conditions support all four as learning-signal quality, and to its measurable counterpart as RPE Fidelity. Because prediction errors shrink as an association is learned, the aim of a well-designed procedure is not to sustain large prediction errors indefinitely, but to keep each reinforcement event informative at the current stage of learning.

RPE Fidelity is conceptually distinct from the well-established construct of procedural fidelity, also termed treatment integrity, which quantifies the extent to which an intervention is delivered as designed and written (Cooper et al., 2020). A reinforcement procedure can be delivered with high procedural fidelity while still providing a poor learning signal, for instance when its schedule has become fully predictable. This distinction corresponds to the difference between M0 and M3 in Section 6.10.

### 6.2 The Neurobehavioral Reinforcement Translation Framework (NRTF)

The Neurobehavioral Reinforcement Translation Framework (NRTF) organizes this evidence across six levels of analysis. It is intended as an integrative heuristic rather than as an independently validated model.

Table 1 summarizes the six levels of the NRTF, the guiding question addressed at each level, and the corresponding evidence base reviewed in this article.

**Table 1:** The six levels of the Neurobehavioral Reinforcement Translation Framework (NRTF).

Level	Guiding question	Evidence base
Neural	What happens in the brain?	Reward prediction error; midbrain dopamine signaling
Synaptic	How does the brain change?	Eligibility trace; dopamine-dependent synaptic plasticity
Computational	How is learning computed?	Actor-critic TD(*λ*); associative and alternative learning models
Behavioral	Which behavior changes?	Contingency; operant reinforcement schedules
Instructional	How does ABA apply these principles?	DTT; NET; token economy
Educational	What outcomes are observed?	Acquisition; maintenance; generalization

### 6.3 An integrative model with feedback loops

Figure 1 traces how information flows across these levels within and across reinforcement events. The pathway is not unidirectional: the way reinforcement is delivered at the instructional level shapes the strength of the neural RPE signal on subsequent trials, and cumulative reinforcement history can, in turn, dampen that signal through satiation or loss of predictability. The figure therefore represents both the feed-forward pathway and the loops connecting the neural, computational, instructional, and educational levels of the NRTF.

**Figure 1:**
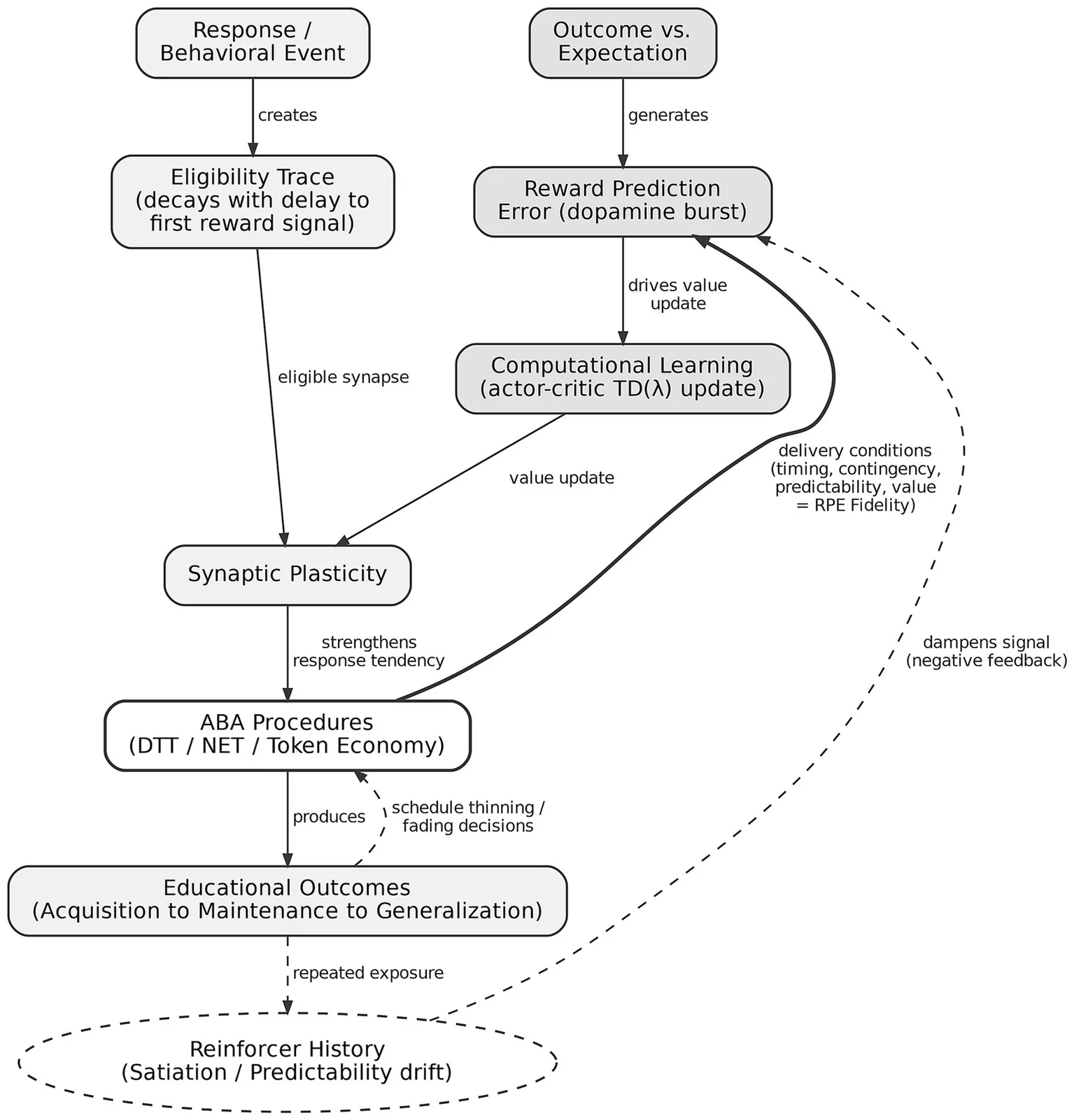
The Neurobehavioral Reinforcement Translation Framework (NRTF): an integrative model with feedback loops. Solid arrows represent the feed-forward translational pathway; dashed arrows represent feedback and modulatory loops. The pathway is a theoretical cross-level synthesis and should not be interpreted as a fully established causal chain in applied settings.

### 6.4 Four core dimensions of RPE Fidelity

I operationalize RPE Fidelity into four dimensions, each corresponding to one of the questions in Section 6.1 and to a term of Equation 5.

Temporal Credit Fidelity concerns the delay *k* between the target response and the first stimulus that reliably signals reward. It draws on the synaptic eligibility trace and its behavioral correlates (Section 3.1), on evidence that knowledge of the response-outcome timeframe modulates the cost of delay (Section 3.2), and on the capacity of conditioned reinforcers to bridge delays (Section 2.3).

Contingency Fidelity concerns the degree to which the outcome is more probable given the target response than in its absence, Δ*P* = *P*(*O*|*R*) − *P*(*O*|¬*R*), and acts through the value of the context. Fiber-photometry recordings in rats show that striatal and midbrain dopamine responses decline when action-outcome or cue-outcome contingencies are degraded (Hart et al., 2024; Garr et al., 2024); neither study manipulated Δ*P* parametrically, so the quantitative form of this relationship remains to be tested.

Prediction Fidelity has two components: whether the outcome remains informative given current expectation, and whether the schedule sustains associability appropriate to the stage of learning. Evidence that phasic and tonic dopaminergic activity differ in their sensitivity to reward predictability (Section 4.1; Hernandez et al., 2008, obtained with intracranial self-stimulation and to be generalized with caution) supports defining this dimension as calibration to the stage of learning rather than as unpredictability.

Outcome Value Fidelity concerns the product of the outcome’s unconditioned value and the learner’s current motivational state, *u*(*o*)·*m*, which motivating operations, satiation, and habituation modify (Laraway et al., 2003; Murphy et al., 2003). Table 2 summarizes the four dimensions.

**Table 2:** The four dimensions of RPE Fidelity, their operational meaning, and the corresponding terms of Equation 5.

Dimension	Guiding question	Operational meaning	Term in Equation 5
Temporal Credit Fidelity	When did the response occur?	Is the delay between the target response and the first stimulus that reliably signals reward short enough, given the stimuli that function as conditioned reinforcers for this learner?	(*γλ*)*^k^*
Contingency Fidelity	What produced this outcome?	Is the outcome clearly more probable given the target response than in its absence, and are any noncontingent outcomes signaled?	*V*(*c*)
Prediction Fidelity	How much does this differ from expectation?	Does the outcome remain informative given current expectation, and does the schedule sustain associability appropriate to the stage of learning?	*δ*, *α_t_*
Outcome Value Fidelity	How much is this worth right now?	Does the reinforcer retain its value given current motivating operations, without signs of satiation or habituation?	*u*(*o*)·*m*

### 6.5 Social reward as a moderator of outcome value

Timing, contingency, prediction, and outcome value are properties of any reinforcement event; social reward is a category of reinforcer, not a property of the same kind, and I therefore treat it as a moderator of outcome value rather than as a fifth dimension. Evidence for a social prediction-error mechanism spans several tiers: in mice, dopamine neurons of the ventral tegmental area encode a social prediction error and drive social reinforcement learning (Solie et al., 2022), and an analogous signal shapes maternal behavior (Xie et al., 2023); in humans, violations of expected social validation modulate midbrain and striatal activity in a manner consistent with prediction error (Poore et al., 2012). This convergence is consistent with social reinforcers entering the same computational pathway as other reinforcers through the outcome-value term; the placement is my own theoretical proposal, not a conclusion the cited studies draw. The dissociation between monetary and social reward described in Section 5.1 is naturally read in the same way, as a moderator of how outcome value is maintained over time. It suggests, as a hypothesis still to be tested (Prediction 4), that pairing tangible reinforcers with praise (Section 6.9) may protect Outcome Value Fidelity against habituation. For learners for whom praise does not yet function as a reinforcer, this placement has further implications, discussed in Section 6.6.

### 6.6 Application to autism and neurodevelopmental conditions

The hypothesis is formulated for reinforcement procedures as they are used in ABA-based intervention for autistic learners and learners with other neurodevelopmental conditions, the population in which these procedures are applied most intensively and systematically. Evidence from general classroom settings (Section 4.2) informs the construct, but the scope of the hypothesis is defined by this population. This has a specific consequence: the parameters of Equation 5 cannot be assumed to take the same values as in the neurotypical samples from which most of the underlying evidence derives. Three differences are directly relevant.

#### 6.6.1 Social and nonsocial reward value

The social motivation hypothesis proposes that social stimuli carry reduced reward value in autism (Chevallier et al., 2012). A meta-analysis of 13 functional imaging studies found atypical striatal responses to both social and nonsocial rewards in autistic participants, together with increased activation to stimuli related to restricted interests, and suggested that differences in sample age may account for some of the heterogeneity in earlier findings (Clements et al., 2018). The interpretation of reduced social orienting as reduced social interest remains debated (Jaswal and Akhtar, 2019), and the evidence concerns group averages rather than individual learners. For the present framework, however, the implication does not depend on how this debate is resolved. The treatment of social reward as a moderator of outcome value (Section 6.5), and the prediction that pairing tangible reinforcers with praise protects outcome value (Prediction 4), both presuppose that praise already functions as a reinforcer. For many autistic learners this is a clinical goal rather than a starting condition, and it is typically addressed through explicit pairing procedures (Dozier et al., 2012). The same presupposition affects Temporal Credit Fidelity: a conditioned reinforcer can bridge the delay to a primary outcome only once it has acquired value (Section 2.3). Which stimulus constitutes the first reliable reward signal is therefore learner-specific, and depends on conditioning history. In this population, Temporal Credit and Outcome Value are coupled through that history, which adds a further, population-specific source of interaction between dimensions.

#### 6.6.2 Estimation of volatility

Bayesian and predictive-processing accounts propose that autistic perception and learning assign different weight to prior expectations relative to incoming evidence (Pellicano and Burr, 2012; Van de Cruys et al., 2014). Consistent with this, autistic adults have been found to overestimate the volatility of their environment, and to show correspondingly reduced physiological surprise when expectations are violated (Lawson et al., 2017). If this pattern extends to reinforcement learning in children, it bears directly on Prediction Fidelity. In the model fitted by Lawson et al., overlearning about volatility was accompanied by reduced learning from probabilistically unexpected events, which were attributed to environmental change rather than treated as informative exceptions. A learner who reads the omissions produced by intermittent schedules, and above all by abrupt transitions between schedules, as evidence that the contingency itself has changed should be more disrupted by them than neurotypical learners. This converges with the latent-cause prediction that the rate of schedule thinning, and not only its endpoint, affects maintenance (Section 6.10). The available evidence derives largely from adult samples and perceptual or decision-making tasks, and its extension to operant learning in children is a hypothesis rather than an established finding.

#### 6.6.3 Individual variation

Both differences are heterogeneous across autistic individuals, and neither can be assumed for a given learner. The practical consequence is that the dimensions of RPE Fidelity must be assessed relative to the individual learner: outcome value through preference assessment and the current motivating operations, temporal credit relative to the stimuli that actually function as conditioned reinforcers for that learner, and prediction relative to that learner’s stage of acquisition. The parameters of Equation 5, including the trace decay λ and the associability weight η, may likewise differ between individuals. Single-case experimental designs, already standard in behavior analysis, are well suited to estimating such parameters at the individual level and to testing whether the predicted interactions hold within learners and not only across groups.

#### 6.6.4 Social validity and learner assent

RPE Fidelity describes the conditions under which reinforcement is more effective; effectiveness is not, by itself, a sufficient criterion for good practice. Behavior analysis has long required that the goals, procedures, and outcomes of intervention be socially valid for the people they affect (Wolf, 1978), and current ethical guidance places increasing emphasis on the learner’s ongoing assent to participation (Behavior Analyst Certification Board, 2020; Breaux and Smith, 2023). Two implications follow for the framework. First, the Outcome Value dimension should be supported through reinforcer variety, choice, and the learner’s own preferences, not through restriction of access intended to raise motivation; a formal account in which motivating operations increase *m* does not license deprivation as a routine means of improving learning. Second, indicators of assent withdrawal, such as attempts to leave the teaching area or consistent refusal, are relevant data for the framework and not only for ethical review: they may signal declining outcome value, and a procedure that produces them cannot be considered high in RPE Fidelity regardless of its other scores. The practitioner checklist (Section 6.9) includes an item reflecting this principle.

### 6.7 A decision aid for reinforcement schedule selection

To make the RPE Fidelity dimensions actionable at the point of clinical decision-making, Figure 2 proposes a decision flowchart linking the developmental status of a target skill to a recommended reinforcement schedule and the dimensions that should be prioritized at each stage. As with the NRTF itself, this flowchart is a clinical heuristic derived from the principles reviewed in Sections 3–5, not an algorithm validated against outcome data. Consistent with Section 6.6 and Prediction 9, transitions toward intermittent reinforcement are recommended to be gradual rather than abrupt.

**Figure 2:**
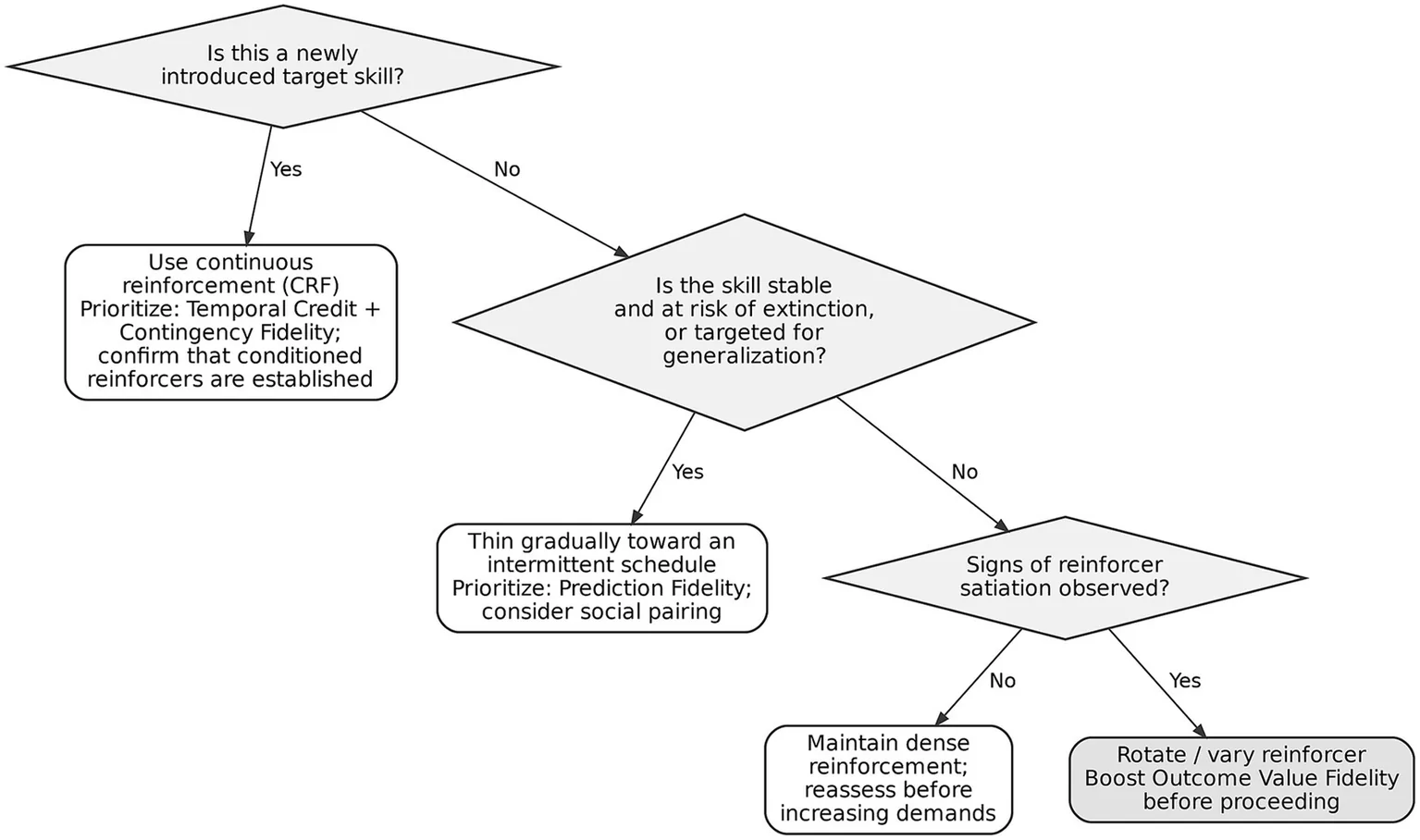
Decision flowchart for reinforcement schedule selection informed by RPE Fidelity dimensions.

### 6.8 Toward a preliminary RPE Fidelity profile

To move the RPE Fidelity construct from a qualitative heuristic toward something researchers could operationalize, I propose a preliminary scoring rubric (Table 3). Each of the four core dimensions is rated on a 3-point scale (0 = absent, 1 = partially met, 2 = fully met). I report the four scores as a profile rather than a sum. The formal account in Section 2.3 predicts that, at the level of the learning update, the dimensions combine multiplicatively, so that a severe deficit in one cannot be compensated by the others; a summed score would misrepresent this structure. Whether the observable ratings show the same multiplicative structure (Prediction 1), and whether they reflect one latent construct or several, are questions for construct validation (Section 7.3).

**Table 3:** Preliminary, non-validated RPE Fidelity profile.

Dimension	0 = Absent	1 = Partial	2 = Full
Temporal Credit Fidelity	Delay to the first reliable reward signal undermines attribution	Attribution possible but not reliable	First reliable reward signal follows the response closely enough for reliable attribution
Contingency Fidelity	Outcome similarly probable with or without the response	Outcome moderately more probable given the response, or noncontingent outcomes unsignaled	Outcome clearly contingent on the response; any noncontingent outcomes signaled
Prediction Fidelity	Outcome fully predicted and uninformative, or unpredictability introduced before the response is established	Partially calibrated to the stage of learning	Well calibrated to the stage of learning
Outcome Value Fidelity	Signs of satiation or habituation given current motivating operations	Some reduction in reinforcer effectiveness	Reinforcer retains full effectiveness

Reported as four separate scores, not summed (see Section 2.3 and Prediction 1).

### 6.9 A practical checklist for practitioners

To translate the NRTF and RPE Fidelity construct into a form immediately usable in clinical and educational practice, I propose the following checklist as a heuristic aid (not a validated assessment instrument) for practitioners designing or reviewing a reinforcement-based teaching program.

#### 6.9.1 Practical checklist: Reward Prediction-Error Fidelity in ABA practice

- Does the first stimulus that reliably signals reward follow the target response closely enough to support attribution?
- Does that stimulus actually function as a reinforcer for this learner (for example, has praise been established as a conditioned reinforcer)?
- Is the reinforcer clearly contingent on the target response, and are any noncontingent reinforcers signaled?
- Is the reinforcer still effective given current motivating operations, without signs of satiation or habituation?
- Is predictability calibrated to the stage of learning, with intermittent reinforcement introduced only once the response is established?
- Is schedule thinning gradual, with a fading plan in place?
- Does the learner continue to assent to participation, and is reinforcer value supported by variety and choice rather than by restricted access?

### 6.10 Competing models and alternative accounts

RPE Fidelity is informative only where its predictions differ from those of existing accounts. Table 4 identifies the principal comparisons, grouped into three classes.

**Table 4:** Competing models and alternative accounts evaluated against the RPE Fidelity hypothesis (M3).

Model	Core claim	Scope
M0: Procedural fidelity	Outcomes depend on correct implementation of the protocol as written	Well established in ABA practice (Cooper et al., 2020)
M1: Quantitative behavioral theory	Outcomes depend on reinforcement rate, delay, and value as described by existing behavioral models	Mathematical Principles of Reinforcement (Killeen, 1994) for response rates; Behavioral Momentum Theory (Nevin and Grace, 2000) for persistence
M2: Alternative learning algorithms	The same delivery variables matter, but combine and scale differently	Attentional (Mackintosh, 1975; Pearce and Hall, 1980), timescale-invariant and retrospective (Gallistel and Gibbon, 2000; Jeong et al., 2022), successor-representation (Dayan, 1993), and Bayesian accounts (Courville et al., 2006)
M3: RPE Fidelity	Delivery conditions determine the learning update through the multiplicative, history-dependent structure of Equation 5	Proposed here; not yet tested

A model based on procedural fidelity alone (M0) cannot explain why two procedures delivered with equally high procedural fidelity can differ in effectiveness when only their delivery conditions vary, since procedural fidelity measures adherence to a protocol, not the informational consequences of how that protocol is executed. A further alternative, that reinforcement procedures work through generic engagement or arousal rather than through reinforcement-specific learning, is not treated as a separate model here, because its most developed formal version is the arousal principle of Killeen's (1994) framework and is therefore tested within M1.

#### 6.10.1 Quantitative behavioral theory (M1)

Killeen’s Mathematical Principles of Reinforcement account for a substantial body of operant behavior through arousal, constraint, and coupling, without reference to neural mechanisms. As noted in Section 2.3, their structure is partly multiplicative and the coupling coefficient plays a role analogous to the eligibility trace; the two accounts diverge mainly in that the Mathematical Principles of Reinforcement describe steady-state response rates, whereas Equation 5 describes trial-by-trial acquisition whose course depends on learning history. Behavioral Momentum Theory addresses a different outcome, persistence, and makes a prediction that directly concerns the Contingency dimension. According to this theory, response rate depends on the response-reinforcer relation, whereas resistance to change depends on the Pavlovian relation between the context and the total rate of reinforcement obtained in it (Nevin and Grace, 2000). Reinforcers delivered independently of the target response, or contingent on an alternative response, should therefore increase persistence even as they reduce response rate, a pattern documented clinically for differential reinforcement of alternative behavior (Mace et al., 2010). Under Equation 5, the same reinforcers raise *V*(*c*) and reduce the update that follows the target response. The two accounts are therefore not in conflict, but they assign the same manipulation opposite effects on different outcomes: M3 predicts slower acquisition, Behavioral Momentum Theory predicts greater persistence. This dissociation implies that tests of RPE Fidelity must measure acquisition and persistence separately, and that analyses of maintenance outcomes (Prediction 5) must control for the context-reinforcer relation specified by Behavioral Momentum Theory.

#### 6.10.2 Attentional models (M2)

Attentional models hold that the rate of learning about a stimulus changes with experience. In Mackintosh's (1975) account, attention increases to stimuli that are reliable predictors of reinforcement; in Pearce and Hall's (1980) account, it increases to stimuli whose consequences remain uncertain. Equation 4 incorporates the Pearce-Hall mechanism, and hybrid models combine both (Le Pelley, 2004). For applied settings, the two mechanisms make different predictions about the Prediction dimension: highly reliable reinforcement during acquisition should strengthen subsequent control by the discriminative stimulus under the Mackintosh mechanism, but reduce the associability of that stimulus under the Pearce-Hall mechanism. A discriminative stimulus trained under reliable reinforcement should therefore support faster learning of a new discrimination under the Mackintosh mechanism, and slower learning under the Pearce-Hall mechanism, than one trained under uncertain reinforcement.

#### 6.10.3 Timescale-invariant and retrospective models (M2)

Rate estimation theory (Gallistel and Gibbon, 2000) and the retrospective causal learning model known as ANCCR (Jeong et al., 2022) both treat the relevant timescale of learning as relative rather than absolute. In ANCCR, the memory trace that allows an earlier event to be identified as the cause of a reward decays with a time constant scaled to the interval between rewards, and mesolimbic dopamine is proposed to convey a causal association signal rather than a prediction error. The behavioral predictions of RPE Fidelity do not depend on dopamine encoding a prediction error, but its neural interpretation does, and results favoring a retrospective account at the neural stage of the research programme (Section 7.3) would require the dimensions to be reinterpreted in those terms. Behaviorally, these accounts preserve the relevance of the Temporal Credit and Contingency dimensions but predict that tolerance for response-reinforcer delay should increase as reinforcement becomes sparser, whereas in Equation 5, with fixed *γ* and *λ*, the cost of a given delay is independent of overall reinforcement density; a factorial manipulation of delay and session-level density, readily implemented within ordinary teaching sessions, separates the two.

#### 6.10.4 Successor-representation models (M2)

The successor representation factorizes value into a learned map of expected future states and a separate estimate of reward at each state (Dayan, 1993; Gershman, 2018). Its distinctive prediction concerns changes in outcome value: a change in reward propagates immediately to well-learned response sequences, whereas a change in the sequence itself does not (Momennejad et al., 2017). Together with the model-free and goal-directed accounts in Section 2.3, the successor representation implies three distinct outcomes for the Outcome Value dimension. After a reduction in *m* (for example, satiation before a session), responding should change immediately under goal-directed control, change immediately for well-practised sequences but not for recently altered ones under the successor representation, and change only after the devalued outcome has been experienced under model-free control.

#### 6.10.5 Bayesian and latent-cause models (M2)

Bayesian accounts treat learning as inference about the structure generating outcomes, with learning rates set by the inferred volatility of the environment rather than by recent prediction error alone (Courville et al., 2006). Latent-cause models add that sufficiently abrupt changes in contingencies can be inferred as the onset of a new context, leaving earlier learning intact rather than modifying it (Gershman et al., 2010); consistent with this, gradual extinction has been found to reduce the later return of extinguished responding relative to abrupt extinction (Gershman et al., 2013). For applied practice, this predicts that the rate of schedule thinning, not only its endpoint, should influence maintenance: abrupt transitions from dense to sparse reinforcement are more likely to be treated as a new context, with consequences for generalization and for the recovery of earlier response patterns. Equation 5 predicts no such effect of transition rate beyond what follows from the gradual change in *V*(*c*) and *α_t_*. Because volatility estimation may differ in autism (Section 6.6), this class of models is also directly relevant to the population in which ABA procedures are most widely used.

Taken together, these comparisons show that the four dimensions are not specific to any single learning algorithm: every model considered here predicts that timing, contingency, predictability, and value matter in some form. What is specific to M3 is the combination rule, the absolute rather than relative scaling of temporal credit, and the dependence of each dimension’s effect on learning history through prediction error. Each of these features can fail independently, and each failure points toward a specific alternative, which is what makes the comparison informative rather than merely confirmatory.

## 7 Discussion

### 7.1 Synthesis

This article has proposed that the effectiveness of a reinforcement procedure depends on the quality of the learning signal its delivery produces, and has formalized this proposal so that its central claim can be tested. The four dimensions of RPE Fidelity correspond to variables that behavior analysis has long recognized; what the formal account adds is a combination rule, under which the dimensions multiply rather than add, and a dependence of their effects on learning history. Placed alongside procedural fidelity, quantitative behavioral theory, and alternative learning models, the hypothesis makes predictions that coincide with these accounts in some respects and diverge from them in others, and the points of divergence define its empirical content.

The literature reviewed here also cautions against treating reinforcement design as a purely mechanical optimization problem. Informativeness that supports learning when calibrated to the stage of acquisition can, if unpredictability is maximized and sustained indefinitely, carry risks analogous to those documented in the behavioral addiction literature (Section 5.2). Withdrawal of extrinsic reinforcement carries a population- and reinforcer-dependent risk of undermining engagement (Section 5.1). Effectiveness itself is not a sufficient criterion of good practice unless the learner’s assent and the social validity of goals and procedures are maintained (Section 6.6).

### 7.2 Testable predictions

#### 7.2.1 Status of the construct and analytic framework

At the level of a teaching procedure, RPE Fidelity is a moderator: it determines how effective a given procedure is on a given occasion. Because the four dimensions are properties of each delivery rather than of the procedure as designed, they enter statistical models as occasion-level predictors of learning, and the moderation claim corresponds to a specific empirical expectation: the dimensions should account for variance in learning outcomes within procedures, including between occasions on which the same procedure is delivered with equal procedural fidelity. The dimensions can first be tested as trial- or session-level predictors in multilevel models of acquisition, with trials nested within sessions and sessions within learners, controlling for procedural fidelity, reinforcer preference, baseline performance, and task difficulty. A stronger test fits Equation 5 and its competitors directly to trial-by-trial data, preferably estimating parameters for each learner, and compares their predictions on held-out observations. Single-case designs standard in behavior analysis, including alternating-treatments and parametric designs, allow both tests within individual learners.

#### 7.2.2 Predictions from the combination rule

##### 7.2.2.1 Prediction 1 (non-compensation)

Holding contingency and predictability constant, a factorial manipulation of the delay to the first reliable reward signal and of outcome value (for example, a highly versus moderately preferred reinforcer, or delivery with and without pre-session access) will produce effects on learning rate that combine multiplicatively. Because a product becomes a sum under a logarithmic transformation, Equation 5 predicts that the two effects will interact on the raw scale of learning rate but will be approximately additive on the logarithmic scale. This result would favor M3. Additivity on the raw scale would favor accounts in which the dimensions contribute independently. Examining both scales also separates a genuine multiplicative combination from floor effects, which can produce apparent interactions on the raw scale alone. Multiplicative combination of reinforcer magnitude and delay is also predicted by hyperbolic discounting accounts of delayed reinforcement (Critchfield and Kollins, 2001) and is compatible with the coupling structure of Killeen's (1994) model. Prediction 1 therefore discriminates M3 from additive, compensatory accounts rather than from M1; the tests against M1 are Predictions 3, 5, and 7. A finer difference concerns the form of the delay function: with a single trace parameter, Equation 5 predicts an exponential decline of the update with delay, whereas discounting accounts predict a hyperbolic decline. Because a mixture of trace time constants can approximate a hyperbolic function, this difference is informative only under the single-parameter assumption.

##### 7.2.2.2 Prediction 2 (contingency through context value)

Noncontingent reinforcement delivered during acquisition slows the acquisition of a target response, an effect already well documented (Rescorla, 1968; Hammond, 1980). The prediction specific to M3 concerns the mechanism. Because contingency acts through the value of the context (Section 2.3), the effect of noncontingent reinforcement should be attenuated when noncontingent deliveries are signaled by a distinct stimulus, which then acquires the value that would otherwise accrue to the context. Attenuation would favor M3. No attenuation would favor accounts in which learning is determined by *ΔP* as a statistical quantity, since signaling leaves *ΔP* unchanged. The basic phenomenon has been reported in animal instrumental learning (Dickinson and Charnock, 1985) but has not been tested in applied settings, where noncontingent reinforcement and the acquisition of new responses frequently co-occur within the same programme.

##### 7.2.2.3 Prediction 3 (stage dependence of predictability)

Holding reinforcement density, outcome value, and contingency constant, reduced predictability introduced before a response is reliably established will impair acquisition, whereas the same reduction introduced once the response is well established will support maintenance and resistance to extinction. A crossover interaction between stage of learning and predictability would favor M3, in which the effect of predictability depends on the learner’s current expectation. An effect in a single direction across stages would favor accounts in which predictability acts through a fixed parameter.

##### 7.2.2.4 Prediction 4 (outcome value and pairing)

Reinforcers showing measurable signs of habituation will be associated with attenuated learning relative to novel or varied reinforcers of comparable nominal value. Pairing a habituating reinforcer with praise will slow this decline, but only for learners for whom praise already functions as a reinforcer, as established by prior reinforcer assessment (Section 6.6). A protective effect of pairing restricted to these learners would favor M3. An effect absent in learners for whom praise is an established reinforcer, or equally present in learners for whom it is not, would count against the placement of social reward as a moderator of outcome value.

##### 7.2.2.5 Prediction 5 (incremental and out-of-sample prediction)

At parity of procedural fidelity, reinforcer magnitude, reinforcement density, and task difficulty, the four dimensions will explain a significant increment of variance in acquisition beyond M0 and M1, and in maintenance beyond M0 and the context-reinforcer relation specified by Behavioral Momentum Theory. In addition, Equation 5, once fitted to trial-by-trial data, will predict held-out learning data more accurately than Killeen's (1994) model. Failure of either test would count directly against the hypothesis.

#### 7.2.3 Predictions distinguishing M3 from alternative learning accounts

##### 7.2.3.1 Prediction 6 (delay and reinforcement density)

A factorial manipulation of response-reinforcer delay and overall session reinforcement density discriminates M3 from timescale-invariant and retrospective accounts (Gallistel and Gibbon, 2000; Jeong et al., 2022). Under M3, with fixed trace parameters, the cost of a given delay will be independent of density. Under the alternative accounts, tolerance for delay will increase as reinforcement becomes sparser.

##### 7.2.3.2 Prediction 7 (acquisition and persistence)

Adding noncontingent or alternative reinforcement to a teaching context will slow the acquisition of the target response (M3) while increasing its resistance to extinction or disruption once established (Behavioral Momentum Theory). The two predictions are compatible, and the test concerns whether measuring both outcomes reveals this dissociation. If added reinforcement failed to slow acquisition when timing and outcome value are held constant, the contingency mechanism of M3 would be contradicted.

##### 7.2.3.3 Prediction 8 (outcome devaluation)

After a reduction in outcome value before a test session conducted in extinction, responding will change immediately under goal-directed control, immediately for well-practised but not for recently modified response sequences under the successor representation, and only after contact with the devalued outcome under model-free control. This prediction constrains the interpretation of M3 rather than testing it against a single rival: the hybrid account specified in Section 2.3 would be supported if the pattern shifts from goal-directed toward model-free with extended training.

##### 7.2.3.4 Prediction 9 (rate of schedule thinning)

Thinning a schedule gradually or abruptly to the same endpoint discriminates M3 from latent-cause accounts (Gershman et al., 2010). Both predict some advantage for gradual thinning, but they differ in what follows a later change of context. Under latent-cause accounts, abrupt thinning leaves the earlier pattern of responding stored rather than modified, so that this pattern should return after a context change more readily than after gradual thinning. Under M3, earlier learning is modified by the prediction errors produced during thinning and no such differential return is expected. Differential return would favor latent-cause accounts, and the effect should be larger for learners who estimate environmental volatility as higher (Section 6.6).

Predictions 1 and 5 provide the primary tests of the hypothesis as a whole; failures on Predictions 6 to 9 instead discriminate among specific alternative accounts.

### 7.3 A staged research programme

Empirical evaluation of RPE Fidelity requires a staged programme, beginning with measurement and behavioral validation before proceeding to neural tests.

#### 7.3.1 Stage 1, measurement and parameter estimation

Develop operational definitions and a coding manual for the four dimensions. Prediction Fidelity requires particular care: because it is defined relative to the stage of learning, it must be scored from the schedule parameters in place and the learner’s documented history rather than from whether learning subsequently occurred, to avoid circularity. This stage should establish inter-rater reliability, examine the factor structure of the ratings, and develop procedures for estimating the parameters of Equation 5 from trial-by-trial data, including tests of parameter recovery on simulated data.

#### 7.3.2 Stage 2, predictive validation

Record the four dimensions during ongoing teaching and test whether they predict rate of acquisition, trials to criterion, retention, maintenance, and generalization, controlling for procedural fidelity, reinforcer preference, baseline performance, and task difficulty (Predictions 1 and 5).

#### 7.3.3 Stage 3, experimental decomposition

Manipulate single dimensions and factorial pairs of dimensions while holding the others constant, including signaled and unsignaled noncontingent reinforcement, to establish causal rather than correlational evidence for each dimension and for their combination (Predictions 1–4).

#### 7.3.4 Stage 4, model comparison

Fit M3 and the alternatives in Table 4 to common trial-by-trial data and compare their predictive performance on held-out data, using the discriminating designs specified in Predictions 5–9.

#### 7.3.5 Stage 5, technological and neural validation

Only after Stages 1 to 4 provide behavioral support for the construct, extend measurement toward physiological and neural correlates, to test whether the dimensions track the hypothesized computations directly rather than only their behavioral consequences. This stage would also test whether dopaminergic signals track prediction errors, as the name of the construct assumes, or causal associations (Section 6.10). Wearable physiological sensors used to estimate engagement (Bustos-Lopez et al., 2022), reinforcement-learning systems already used to personalize instruction for autistic learners (Semi et al., 2026), and generative AI integrated into structured parent-training protocols anchored to an explicit clinical framework, the Autism Open Clinical Model (Morfini, 2023; Morfini and Cesarano, 2025), could eventually support measurement at this stage, but none is a precondition for Stages 1–4.

### 7.4 Limitations

Several limitations qualify the claims made here. Most of the foundational neurobiological evidence on prediction errors and reinforcement timing derives from animal models using simple operant or Pavlovian paradigms, and human evidence from applied settings remains sparse. The interpretation of dopaminergic signals as prediction errors is itself under active revision (Section 2.1), which affects the neural interpretation of the construct, though not its behavioral predictions. Evidence on reward processing and volatility estimation in autism derives largely from adult samples and laboratory tasks (Section 6.6). The overjustification literature specific to learners with intellectual and developmental disabilities remains limited in volume, and estimates of the effectiveness of comprehensive programmes are constrained by the scarcity of randomized designs. Finally, Equation 5 is a deliberately simplified model: it treats each teaching trial as ending with the outcome and does not represent the response chains, prompting procedures, and concurrent targets of ordinary instruction, which will require extensions before the model can be fitted to data from applied settings.

The hypothesis would be substantially weakened, or falsified, by any of the following: additivity of the dimensions on the raw scale rather than multiplicative combination (Prediction 1); absence of incremental or out-of-sample predictive value beyond procedural fidelity and quantitative behavioral theory (Prediction 5); absence of a stage-dependent effect of predictability (Prediction 3); or failure of noncontingent reinforcement to slow acquisition when timing and outcome value are held constant (Prediction 7). The hypothesis thus specifies in advance the pattern of results that would count against it.

### 7.5 Concluding remarks

The central question raised by this article is not whether timing, contingency, predictability, and value matter in reinforcement, which behavior analysis established long ago, but whether a formal account of how they combine, grounded in reinforcement-learning theory, predicts learning better than the accounts already available. If the answer is positive, practitioners would gain a principled way to evaluate not only whether a procedure is delivered as written, but whether each delivery provides the learner with a signal from which learning can occur.

## Data Availability

The original contributions presented in the study are included in the article/supplementary material, further inquiries can be directed to the corresponding author.
